# Analysis of probable lipotoxic damage and myocardial fibrosis in epicardial obesity

**DOI:** 10.18632/aging.203148

**Published:** 2021-06-04

**Authors:** Galina Chumakova, Olesya Gritsenko, Olga Gruzdeva, Yulia Dyleva

**Affiliations:** 1 Federal State Budgetary Scientific Institution Research Institute for Complex Issues of Cardiovascular Diseases, Kemerovo, Russia; 2 Federal State Budgetary Educational Institution of Higher Education, "Altai State Medical University" of the Ministry of Health of the Russian Federation, Barnaul, Russia; 3 Regional State Budgetary Healthcare Institution of Altai Regional Cardiological Dispensary, Barnaul, Russia

**Keywords:** epicardial obesity, cardiac fibrosis markers, myocardial fibrosis

## Abstract

Myocardial fibrosis is considered a key pathological process in the development of cardiovascular diseases. In epicardial obesity (EO), the main cause of fibrosis development is lipotoxic myocardial damage. It is important to detect myocardial fibrosis at an early stage, using non-invasive diagnostic methods. According to the results of echocardiography (ECG), 110 men with general obesity were divided into the following two groups: Group I with epicardial fat thickness (tEAT) ≥ 7 mm (n = 70) and Group II with tEAT < 7 mm (n = 40) without diastolic dysfunction. The levels of metabolic factors, pro-inflammatory cytokines, adipokines, and free fatty acids (FFA), profibrotic markers were determined in both groups. In Group I, the level of interleukin (IL)-6, C-reactive protein, and tumor necrosis factor (TNF)-α increased and that of leptin and adiponectin decreased compared with those in Group II. There was an increase in the level of all studied profibrotic factors in Group I. The level of TNF-α and IL-6 showed a positive correlation with the level of leptin and FFA and a negative correlation with the level of adiponectin. We also observed a relationship between the level of collagen, transforming growth factor (TGF)-β, and metalloproteinase (MMP)-3 and EO. Our results showed that confirmed EO correlates with not only disadipocytosis and increased levels of pro-inflammatory cytokines, but also increased levels of profibrotic factors. This suggests that the studied markers of fibrosis may be used to determine preclinical cardiac fibrosis with lipotoxic myocardial damage in patients with EO.

## INTRODUCTION

Obesity is a chronic disease and leads to the development of numerous complications, which often require an interdisciplinary scientific treatment approach [1]. Obesity is associated with numerous metabolic disorders, including insulin resistance development, increased blood pressure, dyslipidemia, oxidative stress, systemic inflammation, and sympathetic nervous system activation. These disorders affect the morphology and structure of the heart and ultimately its function [2]. The neurohumoral factors controlling these metabolic disorders are produced in white adipose tissues (both subcutaneous and visceral). However, due to the characteristics of innervation, blood supply, and local receptor activities, visceral adipose tissue is accountable for the bulk of specific neurohormones released into the bloodstream [3]. In general, obesity is characterized by chronic inflammation and increased expression and release of proinflammatory neurohumoral factors, including adipokines such as leptin and adiponectin, and proinflammatory cytokines such as tumor necrosis factor-α (TNF-α), interleukin (IL)-1 and IL-6 [4]. Proinflammatory cytokines produced by adipose tissue contribute to both local and systemic proinflammatory responses [5]. The excess adipose tissue in ectopic local fat depots, which include epicardial adipose tissue (EAT), is a source of surplus free fatty acids (FFA), pro-inflammatory cytokines, and adipokines, including leptin, resistin, visfatin, galectin, and omentin [6–10]. However, increased epicardial adipose tissue is associated with a decrease in the synthesis of adiponectin, and this can trigger complex pathophysiological mechanisms resulting in the development of myocardial fibrosis [6–10]. In addition, there is a strong correlation between obesity and leptin resistance (LR), and this results in the development of metabolic syndrome, and, ultimately, the progression of cardiovascular complications. At the core of LR is LR in the hypothalamus and a decrease in the number of leptin receptors [11]. In particular, there is a well-established relationship between obesity and chronic heart failure (CHF) development [12–14]. During obesity, CHF develops as a result of metabolic disorders of the myocardium, and they occur due to visceral obesity [2]. Studies have shown that elevated levels of IL-6, IL-2, and TNF-α are associated with CHF and left ventricle (LV) subclinical dysfunction [3]. Upregulation of these neurohumoral factors contributes to the development of fibrosis and to unfavorable variants of cardiac remodeling [5]. Adipokines play an essential regulatory role in myocardial function and dysfunction given their involvement in myocardial metabolism, myocyte hypertrophy processes, cell death, and extracellular matrix (ECM) structure and composition. A direct regulation of myocardial remodeling components (including matrix metalloproteins (MMPs), tissue metalloproteinase inhibitor, and collagen) by adipokines has been demonstrated in *in-vitro* and *in-vivo* studies [6].

The development of myocardial fibrosis in epicardial obesity (EO) is characterized by progressing diastolic dysfunction, which leads to CHF (when the ejection fraction is retained). In real-world clinical practice, diastolic dysfunction is usually detected by echocardiography (ECG) in a rather late stage. Therefore, it is necessary to search for markers to detect myocardial fibrosis at an earlier stage. The purpose of this study was to study the relationship of EO and neurohumoral activity of EAT with the level of markers of fibrosis.

## RESULTS

### Clinical and anamnestic characteristics of the study patients

Table 1 shows the clinical and anamnestic characteristics of all patients included in the study. The study involved 143 men with obesity of classes I–III, body mass index (BMI) 33.7 ± 3.3 kg/m^2^, and mean age 54.3 ± 8.2 years.

**Table 1:** Clinical and anamnestic characteristics of study patients.

**Parameters**	**Patients, n**	**%**
***Family history***		
Cardiovascular pathology	60	54.5
Type 2 diabetes mellitus	12	10.9
**Anamnesis**		
Smoking	58	52.7
Hypercholesterolemia	63	57.0
Atrial fibrillation	4	3.6
***Comorbidities***		
Chronic bronchitis	17	15.5
Peptic ulcer disease in remission	1	0.9
Chronic cholecystitis	1	0.9
Chronic pyelonephritis	2	1.8
Varicose disease of lower extremities	26	23.6

Two groups of patients were identified, patients with EO (Group I) and patients without EO (Group II). EO was defined as an increase in the epicardial adipose tissue (tEAT) of ≥7 mm, which in clinical research is associated with a higher risk of insulin resistance, dyslipidemia, and other metabolic disorders [15]. The mean tEAT was 8.39 (7.0, 9.0) mm in group I and 4.74 (4.0, 6.0) mm in group II (p < 0.001). The two groups did not differ in age, systolic and diastolic pressures, waist and hip circumferences, and BMI (Table 2).

**Table 2:** Comparative characteristics of patients in groups I and II.

**Parameters**	**Group I (n = 70)**	**Group II (n = 40)**	**p**
Age, years	52.41 (46; 59)	54.71 (50; 59)	0.18
Systolic pressure, mmHg	127.21 (120; 130)	125.32 (120; 130)	0.55
Diastolic pressure, mmHg	81.31 (79; 86)	80.69 (79; 83)	0.31
Waist circumference, cm	106.1 (99; 112)	104.32 (96; 113)	0.34
Hip circumference, cm	111.37 (107; 115)	110.0 (104; 115)	0.40
BMI, kg/m^2^	33.91 (31.41; 34.77)	33.32 (30.85; 35.29)	0.39

Note: p – the level of statistical significance. Abbreviations: BMI– body mass index.

### Characteristics of pro-inflammatory markers, neurohumoral factors, and myocardial fibrosis in patients with EO and those without EO

The levels of pro-inflammatory markers were investigated in all patients. The IL-6, C-reactive protein (CRP), and TNF-α levels were higher in Group I than in Group II; the level of IL-10 was the same in both groups (Table 3).

**Table 3:** Comparative characteristics of pro-inflammatory markers, neurohumoral factors and myocardial fibrosis in groups I and II: med (UQ;LQ).

**Parameters**	**Group I (n = 70)**	**Group II (n = 40)**	**p**
Pro-inflammatory markers			
TNF-α, pg/mL	2.21 (1.21;2.91)	1.24 (1.12;1.34)	<0.001
IL-6, pg/mL	2.42 (2.10;3.41)	1.63 (1.39;1.91)	<0.001
IL-10, pg/mL	8.51 (2.86; 11.92)	8.21 (6.67; 9.23)	0.62
CRP, g/mL	8.29 (5.49;11.12)	4.23 (2.21;5.62)	<0.001
Neurohumoral factors			
SLR, ng/mL	14.61 (11.44;16.42)	22.19 (11.61;30.71)	0.001
Leptin, ng/mL	55.08 (34.87;74.10)	27.52 (19.37;32.23)	<0.001
FLI, r.u.	3.23 (1.59; 4.45)	2.09 (1.39; 2.25)	0.003
Adiponectin, mg/mL	7.61 (4.57;8.52)	11.49 (9.78;12.24)	<0.001
FFA, mmol/L	0.74 (0.49; 0.90)	0.34 (0.28; 0.42)	<0.001
Myocardial fibrosis			
MMP-3, ng/mL	20.52 (13.99;26.74)	11.23 (9.47;14.01)	<0.001
Collagen, pg/mL	38741.29 (34354.41;45524.63)	25368.42 (19823.52;29368.74)	<0.001
TGF-β, ng/mL	49.41 (40.51;58.42)	33.21 (28.23;39.22)	<0.001
VEGFA, pg/mL	69.30 (62.85;87.21)	64.23 (56.25;67.53)	0.001
PICP, pg/mL	741.12 (632.26;821.36)	636.84 (541.85;665.34)	<0.001

Note: p – the level of statistical significance. Abbreviations: IL-6, interleukin-6; IL-10, interleukin-10; CRP, C reactive protein; TNF-α, tumor necrosis factor-α; SLR, soluble leptin receptor; FFA, free fatty acid; FLI, free leptin index; MMP-3, matrix metalloproteinase-3; TGF-β, transforming growth factor-β; VEGFA, vascular endothelial growth factor; PICP, procollagen I C-terminal propeptide.

Furthermore, we evaluated the neurohumoral activity in the epicardial fat. In Group I, the level of adiponectin was significantly lower than that in Group II. In contrast, the level of leptin was higher in Group I than in Group II. We also evaluated the LR parameters in both groups. Free leptin index (FLI) was higher in Group I than in Group II, whereas the soluble leptin receptor (SLR) level was higher in Group II than in Group I. In addition, there was an increase in the FFA levels in Group I (with EO) compared with that in Group II (Table 3). There was a significant correlation between the FLI and tEAT (EO indicator) in Group I (r = 0.33, p = 0.03), but no significant relationship was observed in Group II (r = 0.31, p = 0.11).

The levels of myocardial fibrosis markers (MMP-3, collagen, tumor growth factor (TGF-β), vascular endothelial growth factor (VEGFA), and procollagen I C-terminal propeptide (PICP)) were assessed in patients of both groups. The levels of all myocardial fibrosis markers were significantly higher in Group I (with EO) than in Group II (Table 3).

### Relationships between tEAT and metabolic risk factors in the group of patients with EO

**Figure 1:**
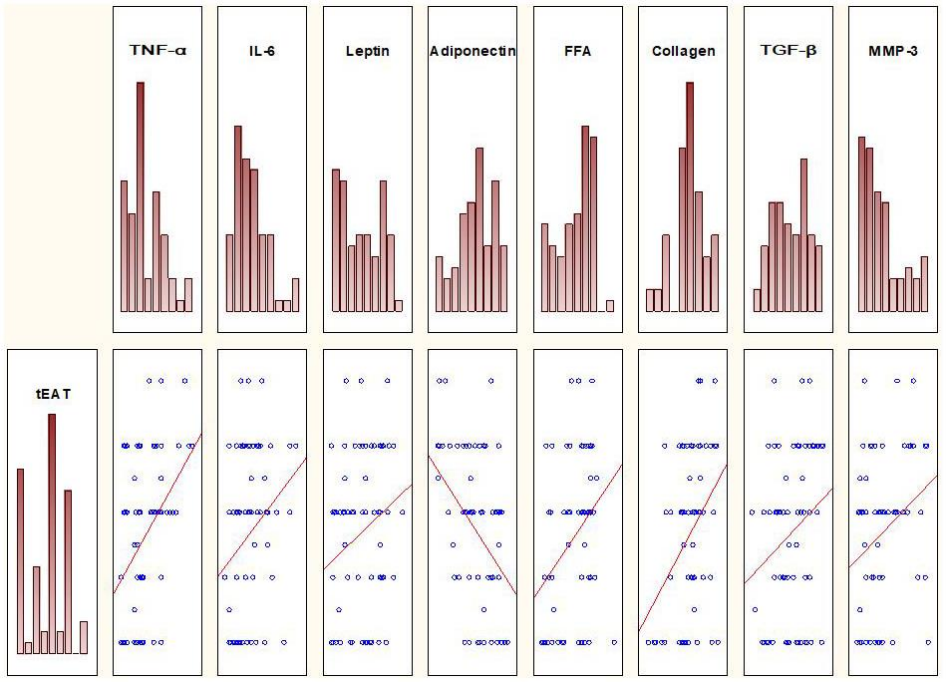
Spearman correlation analysis reveals relationships between tEAT and several metabolic risk factors in the group of patients with EO.

Spearman correlation analysis was performed to study the relationship between metabolic risk factors and tEAT. In Group I, significant positive relationships were observed between EO and the levels of TNF-α (r = 0.31; p = 0.04), IL-6 (r = 0.32; p = 0.03), leptin (r = 0.42; p = 0.003), and FFA (r = 0.30, p = 0.03), and a significant negative relationship with the level of adiponectin (r = -0.31; p = 0.04) (Figure 1).

Spearman correlation analysis was also performed to investigate the relationships between EO and the levels of myocardial fibrosis markers. Significant positive relationships were observed between tEAT and the levels of MMP-3 (r = 0.32, p = 0.047), collagen (r = 0.38, p = 0.017), and TGF-β (r = 0.30, p = 0.04) (Figure 1). No such relationships were observed in Group II. Furthermore, there was no relationship between tEAT and VEGFA and between tEAT and PICP.

### Influence of LR on the levels of myocardial fibrosis markers

The influence of LR on the levels of myocardial fibrosis markers was analyzed using Spearman correlation analysis. In Group I, there was a positive relationship between FLI and VEGFA (r = 0.37, p = 0.03) (Table 4).

**Table 4:** Relationships between levels of adipokines and myocardial fibrosis markers in the group of patients with epicardial obesity.

**Adipokines / Markers of fibrosis**	**MMP-3, ng/mL**	**Collagen, pg/mL**	**TGF-β, ng/mL**	**VEGFA, pg/mL**	**PICP, pg/mL**
FLI, r.u.	r = 0.27 p = 0.19	r = 0.22 p = 0.41	r = 0.10 p = 0.80	**r = 0.37** **p = 0.03**	r = 0.22 p = 0.19
Adiponectin, mg/mL	r = -0.75 p = 0.71	r = -0.18 p = 0.31	r = -0.08 p = 0.67	r = -0.27 p = 0.09	**r = -0.31** **p = 0.03**
Leptin, ng/mL	**r = 0.32** **p = 0.047**	r = 0.21 p = 0.41	r = 0.17 p = 0.34	r = 0.21 p = 0.22	r = 0.26 p = 0.14

Note: p – the level of statistical significance, r is the correlation coefficient. Abbreviations: MMP-3, matrix metalloproteinase-3; TGF-β, transforming growth factor-β; VEGFA, vascular endothelial growth factor; PICP, procollagen I C-terminal propeptide; FLI, free leptin index.

In Group II, there were no relationships between FLI and markers of myocardial fibrosis. Spearman correlation analysis was also performed to assess the effect of adipokines on the levels of myocardial fibrosis markers. There was a negative relationship between the levels of adiponectin and PICP and a positive relationship between the levels of leptin and MMP-3 (Table 4).

## DISCUSSION

Visceral adipose tissue produces a large number of cytokines and adipokines, leading to metabolic and morphological changes. Thus, TNF-α, a widely study cytokine, plays a key role in the pathogenesis and progression of left ventricular remodeling, and its excessive amount leads to myocardial fibrosis and cardiomyocyte apoptosis [4]. It has been shown that TNF-α leads to insulin resistance, which increases the release of FFA [16], the accumulation of which in cardiomyocytes leads to their lipotoxic damage. It has been proved that cytokines as CRPs also play an important role in apoptosis. CRPs stimulate the release of proapoptotic cytokines and inflammatory mediators, which include IL-1 and TNF-α [17]. IL-6, together with TNF-α and IL-1, stimulates the production of proteins in the acute phase of inflammation in the liver and their entry into the general bloodstream. Obesity is associated with an increase in the level of IL-6, whereas weight loss leads to a decrease in its concentration [18]. Here, we studied the effect of leptin on the formation of fibrosis. It has been shown that leptin can cause changes in the ECM, and an increase in its level leads to cardiomyocyte apoptosis and oxidative stress, thereby leading to structural and functional changes in the heart in patients with obesity [19]. It directly stimulates cardiomyocyte hypertrophy and also modulates the fibroblast phenotype by activating the fibrogenic program [20]. The effect of adipokines on the development of myocardial fibrosis has also been studied. The ability of leptin to cause cardiomyocyte apoptosis has been proven in animals. It has been reported that increased cardiomyocyte apoptosis in obese animals compared with that in wild-type mice is due to the changes in leptin signaling (*ob/ob* and *db/db* mice), which is associated with greater DNA damage [19]. The local expression of leptin leads to the development of local oxidative stress, in association with macrophages and lymphocytes, induces proinflammatory cytokines, and activates MMP [21]. Endogenous leptin promotes the remodeling of the ECM by increasing the expression of various compounds such as fibronectin and collagen types I and III [19]. Currently, the mechanisms by which adiponectin contributes to the formation of myocardial fibrosis are actively studied. A previous study showed that adiponectin plays an important role in inhibiting collagen synthesis in the heart, and its level negatively correlated with the level of markers of myocardial fibrosis (PICP and PIIINP) [22]. In addition to these neurohumoral factors, numerous profibrotic factors were considered, including, MMP-3, collagen I, TGF-β, VEGFA, and PICP. The MMPs (the synthesis of which is initiated under the influence of TNF-α, interleukins, and CRP) are involved in the degradation of ECM proteins occurs [23]. MMPs actively participate in the processes of remodeling of the ECM, degrading its components such as collagen, elastin, fibronectin, and glycosaminoglycans [24]. As MMPs play a crucial role in the development of fibrosis, it is considered that they are involved in the formation and progression of CHF. TGF-β leads to myocardial fibrosis by activating the differentiation of fibroblasts to myofibroblasts and accelerating the deposition of ECM components. Moreover, it can inhibit the degradation of the ECM by inhibiting MMPs [25]. It has been demonstrated that TGF-β plays a significant role in the pathogenesis of left ventricular remodeling and the development of CHF [18]. VEGFA is the main regulator of angiogenesis and vascular permeability [26]. Recent study results have shown that VEGFA plays an important role in the control of adipose tissue function and systemic energy metabolism by modulating the adipose tissue vasculature [27]. Obesity is associated with angiogenesis, in which VEGFA plays a key role [28]. An increase in the accumulation of collagen in the cardiac interstitium is a sign of cardiac fibrosis. The synthesis of types I and type III collagen considerably increases during cardiac remodeling, regardless of the etiology of fibrosis [29]. The synthesis and degradation of type I collagen can be indirectly assessed using the circulating biomarkers C-terminal propeptide (PICP) and C-terminal telopeptide (CITP), respectively. Data on the metabolism of collagen, as the main protein of the ECM, are limited [30]. Here, we evaluated the relationship of the neurohumoral activity of EAT with the level of fibrosis markers in patients with EO.

Two groups of patients were identified, patients with EO (Group I) and patients without EO (Group II). EO was defined as an increase in the tEAT of ≥7 mm. In both groups I and II, no relationship was observed between metabolic factors or markers of myocardial fibrosis and BMI. This is due to the fact that BMI reflects the degree of general obesity, in which a significant part of the adipose tissue is represented by subcutaneous fat. Unlike visceral neurohumorally active adipose tissue, subcutaneous fat is relatively inert (does not produce pro-inflammatory neurohumoral factors). This may be the reason that an association between BMI obesity with increased cardiovascular risk has not been elucidated [31]. Here, there was no correlation between BMI and tEAT in Groups I and Group II (r = 0.09, p = 0.45 and r = 0.10, p = 0.40, respectively).

Visceral adipose tissue, including EAT, is a source of pro-inflammatory cytokines, including TNF-α, IL-6, and IL-10 [32]. Pro-inflammatory cytokines TNF-α and IL-1, and CRP, can trigger the synthesis of MMPs [23], leading to an increase in other markers of myocardial fibrosis [33, 34], and together these can activate myocardial fibroblasts. Previous studies have shown that dysadipocytosis could lead to the development of myocardial fibrosis and, subsequently, the disruption of heart diastolic function, which occurs as a result of stiffness caused by the fat “shell,” hypertrophy, and myocardial fibrosis, which leads to CHF [31].

In our study, we demonstrated relationships between tEAT and levels of pro-inflammatory cytokines (TNF-α and IL-6), adipokines (leptin and adiponectin), and markers of myocardial fibrosis (MMP-3, TGF-β, and collagen). We also found that patients with EO develop LR and that it is associated with VEGFA, a marker of fibrosis. Previously, EO was demonstrated to be associated with impaired left ventricular diastolic function [31, 35] in lipotoxic damage. Our findings suggest that elevated levels of profibrotic factors in patients with tEAT ≥ 7 mm may be used as markers of preclinical (and therefore early) signs of myocardial fibrosis, before the detection of diastolic dysfunction in ECG.

There are several limitations in the measurement of tEAT by transthoracic ECG. First, we can only partially measure tEAT by transthoracic ECG. In contrast, both tEAT and volume can be measured by cardiac CT and MRI precisely and more accurately than by ECG. Echocardiographic measurements are not as reproducible as cardiac CT and MRI. Another limitation is the relatively weak inter- and intra-observer variability than those in cardiac MRI and CT. The most important limitation is the lack of specific threshold values to predict pathologies. tEAT appears to increase with age, and it could be influenced by sex and ethnicity. Despite these limitations, tEAT measurement by ECG has the advantage of being an easy-to-perform, readily available, repeatable, and low-cost modality without radiation exposure.

## MATERIALS AND METHODS

### Protocol approval

The study protocol was approved by the Local Ethics Committee of the Regional State Budgetary Healthcare Institution of Altai Regional Cardiological Dispensary and was developed in accordance with the WMA Declaration of Helsinki on Ethical Principles for Medical Research Involving Human Subjects, 2000 edition, and the “GCP Principles in the Russian Federation” approved by the Russian Ministry of Health (2003). All patients provided written informed consent.

### Study population

During 2016–2018, 143 obese male patients attending the Altai Regional Cardiological dispensary were enrolled in this study (mean age, 54.3 ± 8.2 years; obesity class, I–III; mean BMI, 33.7 ± 3.3 kg/m^2^). To exclude coronary atherosclerosis, either coronary angiography (CAG) or multispiral computed tomography of the coronary arteries, according to the indications, was performed in all patients included in the study. The exclusion criteria were a diagnosis of arterial hypertension and diabetes mellitus type 2 diagnoses (as possible causes of the myocardial fibrosis development), and a diagnosis of diastolic dysfunction according to transthoracic ECG. Echocardiography revealed diastolic dysfunction in 33 patients, and they were excluded from the subsequent analysis.

### Anthropometric measurements

Before the study, anthropometric measurements (height and weight of the patients) were performed, and BMI was calculated using the following formula: weight (kg) / height (m^2^). Obesity was diagnosed based on the BMI of more than or equal to 30 kg/m^2^ (RSSC, 2009).

### Assessment of epicardial obesity by ECG

**Figure 2:**
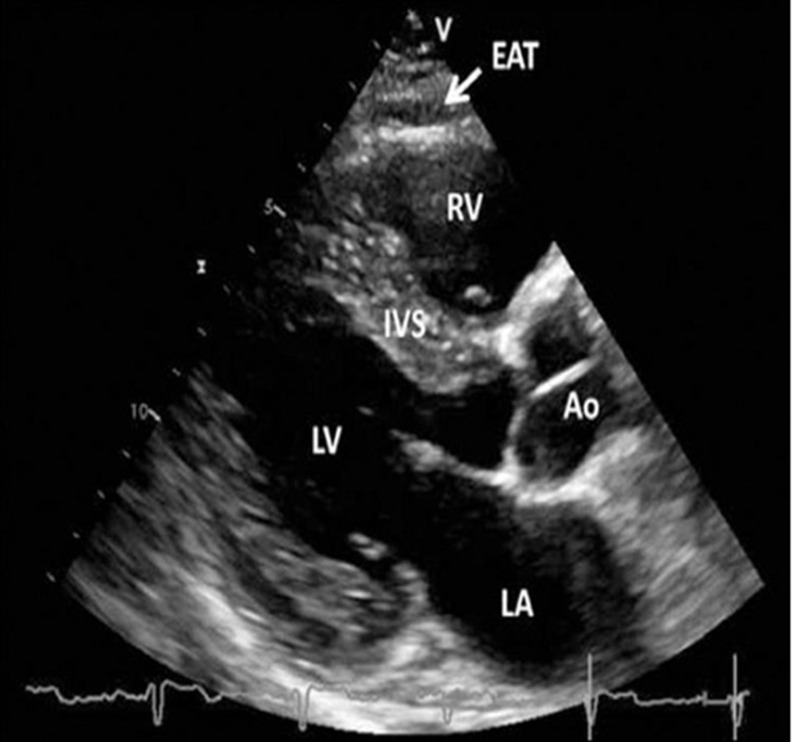
**Transthoracic echocardiographic view of epicardial adipose tissue.** Epicardial adipose tissue is an echo-lucent area between the epicardial surface and parietal pericardium in front of the right ventricular free wall and is indicated with a white arrow. Abbreviations: Ao, aorta; EAT, epicardial adipose tissue; IVS, interventricular septum; LA, left atrium; LV, left ventricle; RV, right ventricle [36, 37].

EO was evaluated by ECG in B-mode using a Vivid 5 ultrasound machine (GE, USA) with a 3.5-MHz mechanical sector sensor. The linear thickness of tEAT was measured in the parasternal position along the longitudinal axis of the left ventricle (LV) behind the free wall of the right ventricle at the end of the systole along the line maximally perpendicular to the fibrous ring of the aortic valve, which was used as an anatomical landmark (Figure 2) [36].

### Multispiral computed tomography (64-slice) of the coronary arteries

Multispiral computed tomography (CT) (64-slice) of the coronary arteries was performed using a multispiral x-ray computed tomographic scanner from Toshiba (Japan), and the data were processed using VITREA and CAG-Philips Integris H3000 (Holland).

### Laboratory assays

### Enzyme-linked immunosorbent assay

The following laboratory parameters were determined in all patients using commercially available enzyme-linked immunosorbent assay (ELISA) kits according to the manufacturer’s recommendations. The serum levels of pro-inflammatory cytokines TNF-α, IL-6, and IL-10, were determined using an ELISA kit obtained from eBioscience (Austria), whereas the CRP level was determined using an ELISA kit obtained from Monobind (USA). The levels of collagen and PICP, two myocardial fibrosis markers, were determined using an ELISA kit obtained from Cloud-Clone Corp. (USA). The levels of other myocardial fibrosis markers (MMP-3, TGF-β, VEGFA) were determined using an ELISA kit obtained from eBioscience (Austria). The adipokine level (adiponectin, SLR) was determined using an ELISA kit obtained from BioVendor (Czech Republic), whereas the leptin level was determined using an ELISA kit from Diagnostics Biochem Canada Inc. (Canada).

### Free leptin index

Leptin sensitivity was evaluated using the FLI, which was defined as the ratio of the total leptin concentration in ng/mL to the soluble leptin receptor SLR concentration in ng/mL. LR was defined as an FLI > 0.25.

### Statistical analyses

Statistical analyses were carried out using STATISTICA 10. For continuous data showing a normal distribution, the mean (M) and standard deviation (SD) are provided; for data with an abnormal distribution, the median (Me) and 25% and 75% quartiles (Me (Q1, Q3)) are presented. The normal distribution hypothesis was tested using Shapiro–Wilk test. We used the non-parametric Mann–Whitney test for the analysis of quantitative data that were not normally distributed. The statistical description of relationships among different parameters was based on Spearman rank correlation coefficient. The level of statistical significance was set at p < 0.05.
